# Anteromedial positioning of the femoral tunnel in anterior cruciate ligament reconstruction is the best option to avoid revision: a single surgeon registry

**DOI:** 10.1186/s40634-020-00225-x

**Published:** 2020-03-07

**Authors:** Ricardo de Paula Leite Cury, Artur Mistieri Simabukuro, Victor de Marques Oliveira, Diego Escudeiro, Pedro Baches Jorge, Fabrício Roberto Severino, Luiz Gabriel Betoni Guglielmetti

**Affiliations:** Orthopedics and Traumatology Department, Faculdade de Ciências Médicas da Santa Casa de Misericórdia de São Paulo, R. Dr. Cesário Mota Júnior, 61 - Vila Buarque, São Paulo, 01221-020 Brazil

**Keywords:** Anterior cruciate ligament, Knee injuries, Anterior cruciate ligament injuries, Anterior cruciate ligament surgery

## Abstract

**Purpose:**

The aim of the study is to compare the risk of revision of single-bundle hamstring anterior cruciate ligament (ACL) reconstruction between the anteromedial, transtibial and outside-in techniques.

**Methods:**

This cohort study was based on data from a single surgeon’s registry. Patients who underwent primary single-bundle ACL reconstruction with hamstring tendon using the anteromedial portal, transtibial and outside-in technique, operated between 1 November 2003 to 31 December 2016, were eligible for inclusion. A minimum follow-up of 2 years was used, and the end-point of the study was revision surgery.

**Results:**

The total number of registered surgeries identified was 665; 109 were excluded, and 556 was the final sample. The overall revision rate was 8.7%. The transtibial technique presented 14/154 [9.9%] of revisions, the transportal 11/96 [11.4%] and the outside-in 22/306 [7.2%]. Separating the outside-in group into central outside-in and anteromedial (AM) outside-in, 18/219 [8.2%] was found for the central outside-in and 4/87 [4.5%] for the AM outside-in technique. Statistical evaluation of the first comparison (transtibial vs. transportal vs. outside-in) obtained *p* = (n.s.) The second comparison (transtibial vs. central transportal vs. central outside-in vs. AM outside-in, *p* = (n.s). Placement was also evaluated: high anteromedial placement (transtibial) vs. central (transportal and central outside-in technique) vs. AM placement (AM outside-in). The high AM placement presented 14/154 [9.9%] of revision, the central placement 29/315 [9.2%] and the AM placement 4/87 [4.5%], *p* = (n.s.) The AM placement was also compared with the other placements (high and central AM), *p* = (n.s.)

**Conclusion:**

Based on the registry of a single surgeon during 14 years of ACL reconstruction, the placement of the femoral tunnel in the high anteromedial region was associated with a rupture rate of 9.9%, central placement with 9.2% and anteromedial placement with 4.5%.

## Background

Reconstruction of the anterior cruciate ligament (ACL) is one of the most performed orthopedic surgeries [1], over 127.000 ACL reconstructive surgeries are performed annually in the USA [2]. The results of this procedure are well documented in several studies, with good to excellent results in approximately 85% to 95% of patients [3, 4]. Despite this rate of success, some questions regarding tunnel placement and tunnel technique continue to be discussed and studied [1, 5, 6].

The femoral tunnel can be drilled using a guide through the tibial tunnel, or by seeking – independent of the tibial tunnel – a point closest to the center of the ACL origin and therefore more similar to the original anatomy, by the out-in technique or by the medial transportal technique. Anatomical studies have shown that the placement of the tunnel using the transtibial technique is not at the center of the ACL origin [6–8], and other biomechanical [6–9] and clinical [10, 11] studies have demonstrated advantages with regard to the stability gained with the more anatomical position of the femoral tunnel.

There are advantages to each technique [1]. The transtibial technique does not require a lateral incision on the distal thigh, an isometric position is obtained, and the femoral tunnel is in the same orientation as the tibial tunnel. The transportal technique includes an anatomical femoral tunnel, independent tunnels, no divergence in the placement of the femoral interference screw and theoretically better rotational stability. The advantages of the outside-in technique are the anatomical positioning of the femoral tunnel, theoretically better rotational stability and lower risk of posterior wall rupture [12]. In the transtibial technique, the placement of the femoral tunnel is dependent of the tibial tunnel, whereas in the medial transportal and outside-in techniques, the surgeon is completely free to choose the placement of the femoral tunnel, regardless of the tibial tunnel. However, this does not guarantee that the placement of the femoral tunnel is better than the positioning achieved with the transtibial technique, as the surgeon may choose inappropriate positions, thus not guaranteeing the superiority of one technique over another [13]. In addition, there is no consensus in the literature as to which technique is associated with more failures, as some studies report a higher rate of revision with the transportal technique [14, 15], whereas others report no differences [5, 16], when comparing anatomical techniques with a transtibial technique.

The present study demonstrates from the experience of a single surgeon the evolution of the surgical technique and the relationship between the femoral tunnel positioning and the ACL reconstruction revision rate.

The objective of the study is to compare the rate of revisions in ACL reconstruction by the transtibial, medial transportal and outside-in techniques and the respective placements of the femoral tunnels sought by the surgeon, using flexor tendon autografts.

## Methods

The study was approved by the ethics and research committee of the Irmandade da Santa Casa de Misericórdia de São Paulo and has the CONEP protocol 15810013.7.0000.5479 registered.

Patient data were extracted from the register of one single surgeon (RPLC). Patients registered for primary ACL reconstruction from 1 November 2003 to 31 December 2016 were eligible for inclusion. Only patients aged 14–50 years who underwent primary single-bundle (SB) ACL reconstruction using a hamstring graft were included. Follow-up started on the date of primary ACL reconstruction and ended with ACL revision surgery. Data on age at index surgery, patient sex, and concomitant injuries noted at index surgery were extracted from the same register. Exclusion criteria were patients with less than 2 years of follow-up, unavailable surgical data, age not between 14 and 50 years, and concomitant lateral or medial ligament lesion requiring repair or reconstruction.

The surgical technique was related to the period in which it was performed. Between November 2003 and May 2010, the transtibial technique was performed; between June 2010 and March 2012, the medial transportal technique was performed; and between April 2012 and December 2016, the outside-in technique was performed.

### Surgical technique

In the transtibial technique, the tibial tunnel was performed with the knee in extension, and a guide in extension was used (65° Howell Guide®; Biomet Sports Medicine Inc., Warsaw, IN, USA). Then, a conventional transtibial guide (aimer) was placed on the posterior margin of the intercondyle. Prior to passing the guide wire (Kirschner 2.4), the aimer was rotated distally to reach a more horizontal position. The graft was passed and fixed with Endobutton® (Smith & Nephew, Andover, MA, USA) on the femur and with a metal interference screw on the tibia. For the transportal technique, the tibial tunnel was performed in the same way as in the transtibial technique, with a guide in extension (65° Howell Guide®; Biomet Sports Medicine Inc., Warsaw, IN, USA). An accessory medial portal was used to visualize the placement of the tunnel. With the knee at 90° of flexion, the center of the native ACL (anatomical positioning) was marked through the conventional anteromedial portal. The femoral fixation was performed with Endobutton® (Smith & Nephew, Andover, MA, USA) and tibial fixation with a metal interference screw. In the outside-in technique, the tibial tunnel was made with the knee in 90° flexion with the tibial guide set at 55°. The femoral tunnel was then made with an anatomical femoral guide. The fixation was made with a metal interference screw from the outside in into the femur and the tibia.

In addition to the different techniques used, the choice of femoral tunnel placement by the surgeon was also changed, and given the importance of this factor, this variable was also evaluated. In the transtibial technique, the surgeon always sought to place the tunnel as low as possible (arthroscopic view with the knee in 90° flexion), turning the transtibial guide at the time the femoral tunnel was made. In the medial transportal and outside-in techniques, the surgeon tried to perform the anatomical reconstruction placing the femoral tunnel at the center of the ACL in order to improve rotational control of the knee. In July 2015, the surgeon was performing the outside-in technique, seeking to place the femoral tunnel at the center of the ACL, and therefore began placing it at the center of the AM band. To standardize the evaluation, the techniques were termed: transtibial, central transportal, central outside-in (surgeries performed between March 2012 and July 2015) and anteromedial outside-in (surgeries performed between July 2015 and December 2016). Therefore, the need for revision was assessed as follows to compare the techniques: transtibial x medial transportal x outside-in. In addition, it was also evaluated considering the placement of the femoral tunnel: transtibial (high anteromedial) x central transport x central from outside to inside x anteromedial from outside to inside (AM).

All groups received the same rehabilitation protocol, with use of crutches for 2 weeks, without postoperative immobilization, release for quadriceps open kinetic chain exercise after the eighth week, running after the sixteenth week, and return to sports after 6 months after proprioceptive evaluation and strength tests applied by the physical therapist.

### Statistical analysis

Data were subjected to statistical analysis by a professional hired in the program SPSS, version 13.0 for Windows. For descriptive analysis, qualitative variables are expressed as frequencies (number and percentage) and visually. The quantitative variables are expressed with summary measures (mean, median, standard deviation, minimum and maximum). For qualitative versus qualitative inferences, the chi-squared or Fisher’s exact test was used. For qualitative versus quantitative inferences, Student’s t-test (parametric) or the Mann-Whitney test (nonparametric) was used at a 5% significance level. The sample number was calculated according to literature examples.

## Results

Of the 665 operated patients, 109 were excluded due to the exclusion criteria, and the final sample size was thus 556 patients. Of these, 461 were male [82.9%] and 95 were female [17.1%]. The mean age at surgery was 24.4 years [14-50] and the mean follow-up was 4.36 years [1,5-8,5]. Associated meniscal injuries were identified in 255 patients [45.8%]. Revision indication occurred in 47 of the 556 patients evaluated [8.7%] (Table 1).

**Table 1:** Demographic data of the study population

Variable	Total	Transtibial	Transportal	Outside-in
Age	24.4 years (14–50)	22.3 years (16–44)	24.4 years (14–50)	26,5 years (16–48)
Gender	Male: 461, Female: 95	Male: 102, Female: 52	Male: 75, Female: 21	Male: 284, Female: 22
Follow-up	4.36 years (1,5–8.5)	5.3 years (1,8–8.5)	4.2 years (2–6.4)	3.3 years (1.5–7.2)

The relationship of performance of revision with sex and age was evaluated. Regarding sex and age, no difference was observed with regard to performance of revision (*P* = n.s.).

**Figure 1:**
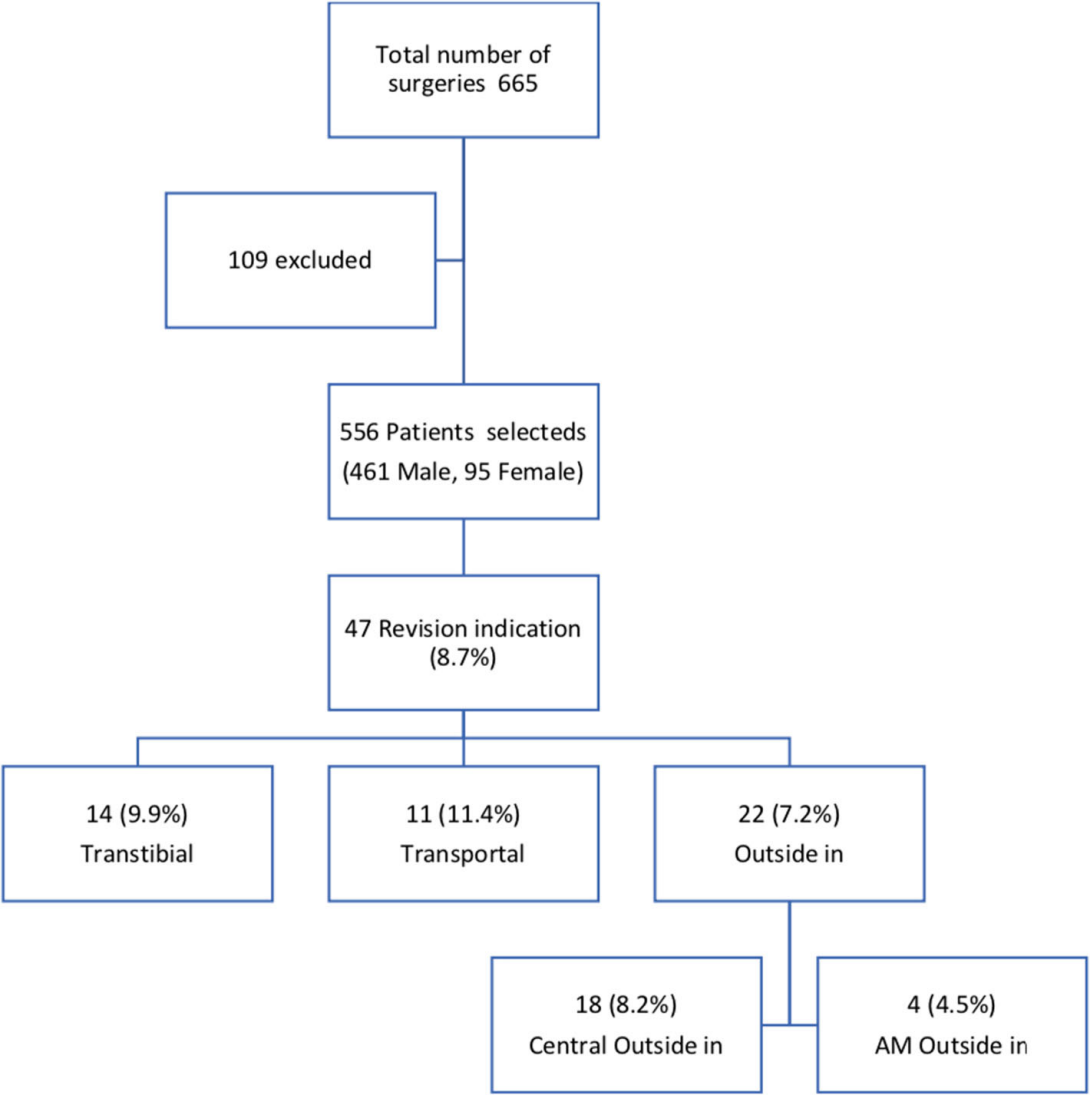
Flow diagram of the study

The relationship between performance of revision and surgical technique employed and femoral tunnel placement was evaluated. Revision indication occurred in 47 patients. Of these, 14 occurred in patients subjected to the transtibial technique with a mean follow-up of 5.3 years, 11 in patients subjected to the transportal technique who had a mean follow-up of 4.2 years and 22 in patients subjected to the outside-in technique who presented an mean follow-up of 3.3 years. Separating the outside-in technique into anatomical outside-in and AM outside-in, 18 revisions occurred with the anatomical outside-in and four with the AM outside-in technique. The transtibial technique presented 14/154 [9.9%] of revisions, the transportal 11/96 [11.4%] and the outside-in 22/306 [7.2%]. Separating the outside-in group into central outside-in and AM outside-in, 18/219 [8.2%] was found for the central outside-in and 4/87 [4.5%] for the AM outside-in technique (Fig. 1).

Statistical evaluation of the first comparison (transtibial X transportal X outside-in), using the chi-square test, obtained no statistical significance. The second comparison (transtibial x central transportal x central outside-in x AM outside-in), applying the chi-square test, obtained no statistical significance (Table 2).

**Table 2:** Relationship between performance of the review and surgical technique employed and placement of the femoral tunnel using the chi-square test

TT X TP X OI	*p* = 0,4*
TT X TP X cOI X amOI	*p* = 0,409*
TT X CENTRAL (TP + cOI) X amOI	*p* = 0,371*
(TT + CENTRAL) X amOI	*p* = 0,208*

*TT* transtibial, *TP* Transportal, *OI* Outside-In, *cOI* Central Outside-In, *amOI* AM Outside-in, CENTRAL (TP + cOI). * P > 0.005

Placement was also evaluated: high anteromedial placement (transtibial) X central (transportal and central outside-in technique) X AM placement (AM outside-in). The high AM placement presented 14/154 [9.9%] of revision, the central placement 29/315 [9.2%] and the AM placement 4/87 [4.5%]. Applying the chi-square test, *p* = (n.s.) was obtained, i.e., no statistical significance. The AM placement was also compared with the other placements (high and central AM). The AM placement had a 4/87 [4.5%] rate of reruptures, while the others had 43/469 [9.2%]. Applying the chi-square test, *p* = (n.s.) was obtained, with no statistical significance.

Regarding surgical complications, there were two cases of superficial infection (transtibial group) treated only with antibiotic therapy, progressing to healing, and one case of arthrofibrosis (transportal group) that required arthroscopy and manipulation under anesthesia, progressing to total gain of mobility.

## Discussion

The most important finding of the present study was that the anteromedial positioning of the femoral tunnel has a lower revision rate in ACL reconstruction.

It is not known where the best point is to the center the femoral tunnel [17–19].

Two similar studies published in 2013 [20] and 2015 [21] evaluated the mechanical functions of AM and posterolateral (PL) bands. Both studies found that the AM band presents greater anteroposterior and rotational stability. Considering therefore that the anteromedial region or band is the main stabilizer in both the anteroposterior and rotational planes, perhaps the ideal point for the center of the femoral tunnel is not exactly in the center of the LCA, as demonstrated in the previously mentioned studies [15, 19]. Another interesting recent discussion is related to the presence of two types of fibers in the ACL: direct and indirect [22]. In 2012, a histological study [23] pointed to a microscopic four-layer structure in both the direct femoral and tibial insertion, demonstrating that the direct fibers are stronger and biomechanically more important, located in the lateral femoral intercondylar ridge. In addition, a recent study [24] demonstrated that no area of the ACL is actually isometric, but the region anterior to the center (between the AM and PL bands), i.e., the lateral femoral intercondylar crest, is the most isometric. Therefore, when approaching the center of the tunnel to lateral femoral intercondylar ridge and the anteromedial region, the center of the tunnel approaches a point that may function mechanically better than in the center of the native ACL, and this positioning is usually close to that obtained by transtibial reconstruction.

Studies that report more reruptures with the medial transportal technique compared to the transtibial technique are retrospective and have several limitations. The first important study that presented these results was published in 2013 [15]. This was an evaluation of medical records of the Danish registry of ACL reconstructions, in which 9239 reconstructions performed by several surgeons in several hospitals between 2007 and 2010 were evaluated, a period considered by the study of popularization of the transportal technique, while the transtibial technique was already well-established. In addition, only the technique performed was evaluated, and not the exact placement of the tunnel, as there was no imaging examination to verify where the tunnels were actually placed. Another important study [14], with a similar methodology, evaluated the Swedish registry and found more reruptures with the medial transportal technique compared to the transtibial technique, but the authors subdivided the patients of the medial transportal group into anatomical transportal and reference transportal. For this division, the authors classified as anatomical those surgeries that the surgeons reported to have performed only anatomically, and classified as reference those who reported in the questionnaire to have used some anatomical parameters and the medial portal to choose the placement of the femoral tunnel. When comparing the transportal group with the transtibial group, they found the same need for revision in the groups. These studies [14, 15] are usually cited, but there are also other publications showing superior clinical results with the transportal technique, with less need for revision or without difference when comparing the medial transportal and transtibial techniques [5, 16]. It is important to note that we did not find any prospective, randomized, controlled series with homogeneous groups and patients operated by the same surgeon, that found more reruptures or better clinical outcome in the transtibial group.

In the present study, when comparing the techniques (transtibial x medial transportal x outside-in), the rerupture rate was 9.9% for the transtibial, 11.4% for the medial transportal and 7.2% for the outside-in technique, with no significant difference. However, comparing techniques is different from comparing tunnel placement. The present study compared the rerupture rate of the techniques but also compared the rerupture rate related to the placement sought by the same surgeon over 14 years. Two other recently published studies compared the rerupture rate related to the placement of the femoral tunnel. In the first [25], a rerupture rate of 7% was found in patients in whom the ACL was placed in the central (mid-bundle) position, and of 1.8% in those in the anteromedial bundle footprint position, with statistical significance. The second study [26] compared the anteromedial position obtained by the transtibial technique with the central position obtained by the medial transportal technique and found a rerupture rate of 5.9% of the cases in the anteromedial position versus 6.9% in the central region. In the present study, when comparing high AM (transtibial) x central x AM placement, there was a 9.9% rate of rerupture with high AM, 9.2% with central, and 4.5% with AM. Although the *P*-value of 0.371 indicates no significant difference, we believe this is a type 2 statistical error, i.e., with a larger sample, the *p*-value would tend to decrease until at some point it reaches statistical significance. When comparing the AM placement with the others, the AM placement had a 4.5% rerupture rate, while the others had 9.2%, also with no statistical significance, but perhaps with the same type 2 error, corroborating with the data in the literature presented above and with the greater importance of the anteromedial region of the ACL [20, 21].

### Study limitations

Despite evaluating the placement of the tunnels with the different techniques, this placement was not documented with imaging tests. In addition, the techniques changed over time, and thus older techniques had more time for the occurrence of new ruptures. Another factor that deserves to be mentioned is that the transportal technique has a smaller sample than the other techniques, and thus few cases of rerupture associated with this technique considerably change the rate in this group.

Knowing that the best results are obtained with the anteromedial positioning of the femoral tunnel in the ACL reconstruction. The choice of technique by the surgeon in daily practice is made easier, and the risk of revisions decreases.

## Conclusions

Femoral tunnel placement in the upper anteromedial region was associated with a rupture rate of 9.9%, central placement with 9.2%, and anteromedial placement with 4.5%. Therefore, the tunnel placed in the anteromedial position is the best option to avoid revision of the ACL reconstruction.

## Data Availability

The datasets used and/or analysed during the current study are available from the corresponding author on reasonable request.

## Abbreviations

ACL: Anterior cruciate ligament
AM: Anteromedial
PL: Posterolateral
RPLC: Register of one single surgeon
SB: Single-bundle
