# Correction to: Anteromedial positioning of the femoral tunnel in Anterior Cruciate Ligament reconstruction is the best option to avoid revision: a single surgeon registry

**DOI:** 10.1186/s40634-020-00249-3

**Published:** 2020-05-16

**Authors:** Ricardo de Paula Leite Cury, Artur Mistieri Simabukuro, Victor de Marques Oliveira, Diego Escudeiro, Pedro Baches Jorge, Fabrício Roberto Severino, Luiz Gabriel Betoni Guglielmetti

**Affiliations:** Orthopedics and Traumatology Department, Faculdade de Ciências Médicas da Santa Casa de Misericórdia de São Paulo, R. Dr. Cesário Mota Júnior, 61 - Vila Buarque, São Paulo, 01221–020 Brazil

**Correction to: J Exp Orthop 7:11 (2020)**

**https://doi.org/10.1186/s40634-020-00225-x**

Following publication of the original article, the authors opted to correct the middle name of author Ricardo de Paula Leite Cury from Paulo to Paula.

The original article [1] has been updated.
